# Design, synthesis, and biological evaluation of symmetrical azine derivatives as novel tyrosinase inhibitors

**DOI:** 10.1186/s13065-021-00780-z

**Published:** 2021-09-29

**Authors:** Somaye Karimian, Fatemeh Kazemi, Mahshid Attarroshan, Maryam Gholampour, Shiva Hemmati, Amirhossein Sakhteman, Yasaman Behzadipour, Maryam Kabiri, Aida Iraji, Mehdi Khoshneviszadeh

**Affiliations:** 1 Department of Medicinal Chemistry, School of Pharmacy, Shiraz University of Medical Sciences, Shiraz, Iran; 2 Medicinal and Natural Products Chemistry Research Center, Shiraz University of Medical Sciences, Shiraz, Iran; 3 Pharmaceutical Sciences Research Center, Shiraz University of Medical Sciences, Shiraz, Iran; 4 Department of Pharmaceutical Biotechnology, School of Pharmacy, Shiraz University of Medical Sciences, Shiraz, Iran; 5 Biotechnology Research Center, Shiraz University of Medical Sciences, Shiraz, Iran; 6 Stem Cells Technology Research Center, Shiraz University of Medical Sciences, Shiraz, Iran; 7 Central Research Laboratory, Shiraz University of Medical Sciences, Shiraz, Iran

**Keywords:** Melanin, Azine derivatives, Tyrosinase inhibitors, Molecular docking

## Abstract

A series of symmetrical azine derivatives containing different substituted benzyl moieties were designed, synthesized, and evaluated for their inhibitory activity against tyrosinase. The results showed that compounds **3e**, **3f**, **3h**, **3i**, **3j,** and **3k** possess effective tyrosinase inhibition with IC_50_ values ranging from 7.30 μM to 62.60 μM. Particularly, compounds **3f** displayed around three-fold improvement in the potency (IC_50_ = 7.30 ± 1.15 μM) compared to that of kojic acid (IC_50_ = 20.24 ± 2.28 μM) as the positive control. Kinetic study of compound **3f** confirmed uncompetitive inhibitory activity towards tyrosinase indicating that it can bind to enzyme–substrate complex. Next, molecular docking analysis was performed to study the interactions and binding mode of the most potent compound **3f** in the tyrosinase active site. Besides, the cytotoxicity of **3f**, as well as its potency to reduce the melanin content were also measured on invasive melanoma B16F10 cell line. Also, **3f** exhibited above 82% cell viability in the A375 cell line at 10 µM. Consequently, compounds **3f** could be introduced as a potent tyrosinase inhibitor that might be a promising candidate in the cosmetics, medicine, and food industry.

## Introduction

Melanin is known as a major pigment found in the eyes, hair, and skin of animals and humans which has protective roles against the harmful effects of ultraviolet (UV) irradiation, oxidative stress, DNA damage, and malignant transformation [1, 2]. Despite the key features of melanin, excessive production, and hyperpigmentation of melanin cause dermatological disorders such as melasma, ephelides, chloasma, freckles, melanoderma, and senile lentigines [3]. The excess melanin synthesis can also induce inflammation such as eczema, irritant and allergic eczema contact dermatitis, which may be attributed to critical and emotionally distressing difficulty [4]. Moreover, there is some evidence about the correlation between neuromelanin and the pathogenesis of Parkinson’s disease [5]. Also in the agricultural industry, overproduction of melanin in fruits and vegetables causes food browning and decline in product quality [6].

**Fig. 1:**
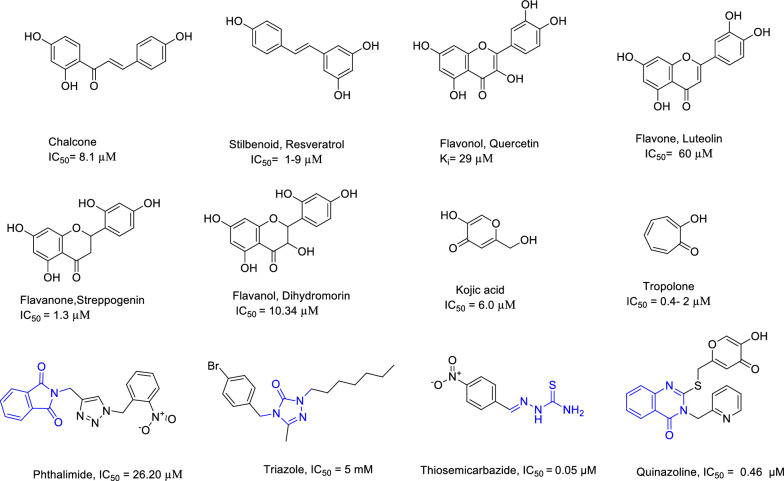
Chemical structures of some tyrosinase inhibitors from natural or synthetic sources

Tyrosinase (Polyphenol oxidase, a copper-containing enzyme, PPO, E.C.1.14.18.1) is a critical rate-limiting enzyme in the melanogenesis pathway. Tyrosinase plays a central role in the biosynthesis pathway of melanin pigment by catalyzing the hydroxylation and oxidation of monophenols to *o*-diphenols (monophenolase activity) and the oxidation of *o*-diphenols to *o*-quinones (diphenolase activity) [7]. To get rid of the mentioned undesirable problems, searching for novel and effective tyrosinase inhibitors is highly demanded. Although a large number of tyrosinase inhibitors have been discovered [8] such as chalcones [9], stilbenes [10–13], flavonol [14], flavone [15, 16], flavanone [17], flavanol [18], kojic acid [19–23], tropolone [8, 24–27], phthalimide [28], triazole [29] thiosemicarbazide [30] and quinazoline [31] derivatives (Fig. 1), only a few of them have been applied to the market due to their harmful and undesirable side effects, such as lack of safety, low efficacy and allergenic reactions [32, 33]. Consequently, it is necessary to search for new and potent tyrosinase inhibitors with fewer adverse effects.

Azines, N–N linked diamines are useful and stable compounds with interesting chemical attributes. Recently, azine-containing compounds gain lots of attention as important scaffold for drug designing owing to their modulating behavior towards bio-receptors [34, 35]. Azines also demonstrated antibacterial, antimalarial, antiviral, antitumor, and anti-inflammatory properties [36, 37].

As a result, in this study, novel series of azine derivatives were rationally designed, synthesized, and evaluated against the tyrosinase enzyme. Molecular docking analysis of the most potent derivative was also obtained to achieve a distinct insight into the binding mode and interactions of the compounds in the tyrosinase enzyme active site. Moreover, the kinetic, cytotoxic, as well as melanin content assays, were also performed.

## Results and discussion

### Design approach

Resveratrol (3,5,4′-trihydroxy-*trans*-stilbene) (**A**) is a natural antioxidant with numerous beneficial effects on human health such as neuroprotective, cardioprotective, anti‐inflammatory, and anti-cancer effects which prompted the use of resveratrol as a therapeutic agent [38, 39]. Resveratrol is also known as a powerful tyrosinase inhibitor with an IC_50_ value of 26.63 μM with no marked toxicity (main concern at doses of ≥ 0.5 g/day for long-term use) [40].

**Fig. 2:**
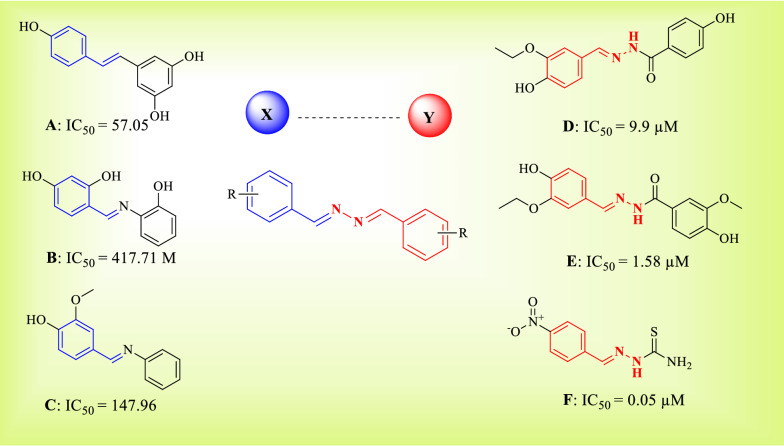
Molecular hybridization and fragment-based approach for designing of bis-aryl hydrazine derivatives as tyrosinase inhibitors

Oozeki et al. reported resveratrol base structure as strong tyrosinase inhibitors with an IC_50_ value of 0.37 μM for the most potent compound (compound **B**, Fig. 2). Their results showed that the symmetry bibenzyl skeleton could easily bind to the active site of the tyrosinase and improved the inhibitory potency [41]. Furthermore, incorporation of an azo group into the linker while keeping bi-aryl structure gave azo-resveratrol derivatives**.** Particularly, compound **C** showed IC_50_ = 36.28 ± 0.72 μM in a dose-dependent manner, comparable to that of resveratrol [42].

Recently, it has been reported that some hydrazine-containing compounds, such as compounds **D** [43], **E** [44], and **F** [45], were effective tyrosinase inhibitors with an IC_50_ value of 9.9, 1.58, and 0.05 μM, respectively [46, 47]. Considering these structural features of all depicted compounds, most of them contain the same pharmacophore so that a linker with optimum length (two to four atoms) is connected to two aromatic rings (Ring X-Linker-Ring Y).

As a result, molecular hybridization and fragment-based drug design strategy were performed to develop a series of 1,2-bisaryl hydrazine derivatives as tyrosinase inhibitors. Various substituents were performed on benzylidene moieties to define beneficial structure–activity relationships (SARs).

### Chemistry

**Scheme 1:**
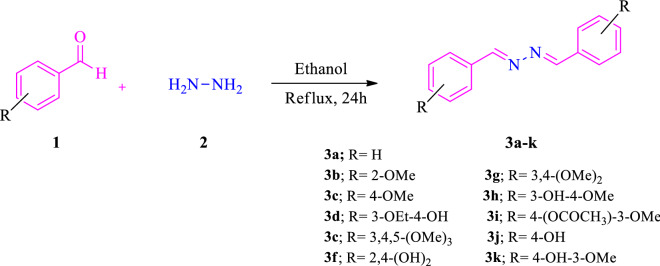
The synthetic route for the synthesis of bis aryl hydrazine derivatives (**3a**–**k**)

A series of bis aryl hydrazine hybrids (**3a**–**k)** was synthesized according to the general synthetic route depicted in Scheme 1. Commercially available aryl aldehydes (**1**) were reacted with hydrazine hydrate (**2**) in refluxing ethanol for 24 h to give the corresponding compounds (**3a**–**k**). After cooling, the precipitate was filtered, washed with cold water, and recrystallized in methanol. The structures were fully characterized and confirmed by IR, ^1^H-NMR, ^13^C-NMR, MS, and elemental analysis.

### Tyrosinase inhibitory activity

The inhibitory effects of all synthesized derivatives (**3a**–**k**) on tyrosinase are presented in terms of IC_50_ in Table 1. Kojic acid was used as a positive control for comparative purposes. In general, six compounds **3e**, **3f**, **3h**, **3i**, **3j**, and **3k** showed considerable inhibitory effects on tyrosinase with IC_50_ ranging from 7.3 to 62.6 μM.

**Table 1:** Tyrosinase inhibitory effects of synthesized compounds **3a**–**j** in comparison with kojic acid and Binding energy
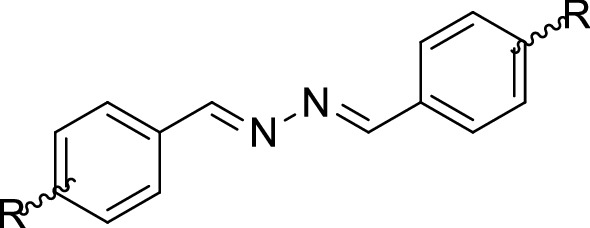

Tyrosinase inhibitory activity^a^		
Compounds	R	IC_50_ (µM)^b^
**3a**	H	> 100
**3b**	2-OMe	> 100
**3c**	4-OMe	> 100
**3d**	3-OEt-4-OH	> 100
**3e**	3,4,5-(OMe)_3_	20.10 ± 0.01
**3f**	2,4-(OH)_2_	7.30 ± 1.15
**3g**	3,4-(OMe)_2_	> 100
**3h**	3-OH-4-OMe	57.34 ± 0.02
**3i**	4-(OCOCH_3_)-3-OMe	28.11 ± 0.52
**3j**	4-OH	62.60 ± 0.71
**3k**	4-OH-3-OMe	12.90 ± 0.18
**Kojic acid**	–	20.24 ± 2.28

^a^Values represent means ± SE of 3–6 independent experiments

^b^50% inhibitory concentration (IC_50_)

^c^Not determined

The unsubstituted benzyl derivative (**3a**) had no significant inhibitory activity at the tested concentrations (IC_50_ > 100 μM).

To investigate the effect of the substituted moiety, different groups were introduced on the benzyl pendant. In the case of mono-substitution, it was found that the presence of one methoxy at different positions of benzyl ring did not show any improvement in the inhibitory activity (**3b**, R = *ortho*-OCH_3_ with IC_50_ > 100 μM and **3c**, R = *para*-OCH_3_ with IC_50_ > 100 μM) compared to unsubstituted one. The isosteric replacement of methoxy at *para* position (**3c**), with a hydroxyl group in compound **3j**, increased the inhibitory effect against tyrosinase (IC_50_ = 62.6 ± 0.71 µM).

Besides, compared to mono substitutions, multi-substitutions on benzyl ring significantly enhanced inhibitory potency as can be seen in compounds **3e**, **3f**, **3h**, **3i,** and **3k**. The exception in this series came back to the compounds **3d** (R = 3-OEt-4-OH, IC_50_ > 100 μM) and **3g** (R = 3,4-(OMe)_2,_ IC_50_ > 100 μM) which depicted weak anti-tyrosinase activity. Particularly, the most potent ligand identified in this study was compound **3f** bearing 2,4-dihydroxy moiety with IC_50_ = 7.30 ± 1.15 µM, followed by **3k** (R = 3-OMe-4-OH) with an IC_50_ value of 12.90 ± 0.18 μM. On the other hand, the substitution of a *para* hydroxyl group in compound **3k** (R = 3-OMe-4-OH, IC_50_ = 12.9 ± 0.18 μM) with an acetoxy moiety resulted in **3i** which showed a significant decrease in the inhibitory effect (IC_50_ = 28.1 µM). From the screening data, **3e** containing 3,4,5-trimethoxy substituted groups at R known as the third potent compound in this series (IC_50_ = 20.10 ± 0.01 µM) with approximately the same value of potency compared to that of the standard kojic acid with an IC_50_ value of 20.24 ± 2.28 μM.

### Kinetic studies

**Fig. 3:**
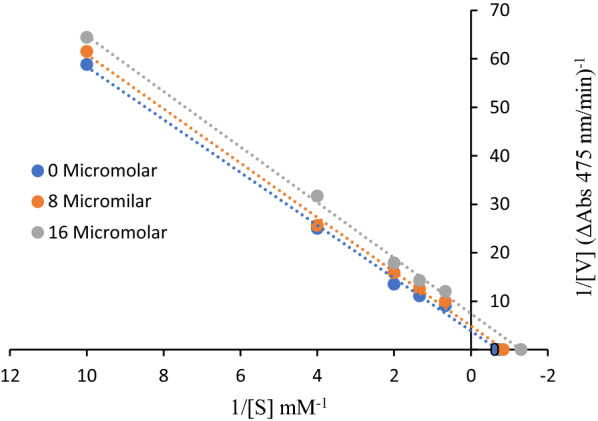
Lineweaver–Burk plot of mushroom tyrosinase enzyme inhibition by different concentrations of **3f** in the presence of L-DOPA as a substrate. The reciprocal tyrosinase inhibitory activity was plotted against the reciprocal substrate concentration (double reciprocal plot, n = 3). K_m_ is the Michaelis–Menten constant and V_max_ is the maximum reaction velocity

Kinetic studies were performed to examine the mechanism and type of inhibition by compound **3f** as the most potent derivative against tyrosinase. The results are presented in Fig. 3. Lineweaver–Burk plots (plot of 1/V versus 1/[S]) for the inhibition of tyrosinase were obtained with several concentrations of **3f** as the inhibitor and L-DOPA as the substrate. The analysis showed that V_max_ and K_m_ values decreased in the presence of increasing concentrations of compound **3f**. With these results, it can presume that the type of inhibition for compound **3f** is uncompetitive.

### Molecular docking study

The molecular binding analysis was then performed to gain an understanding of the interactions and binding mode of **3f** in the tyrosinase active site. The results are presented in Table 2. Tyrosinase has H_2_L_2_ tetramer structure with two H subunits and two L subunits. The active site placed in the H subunit comprises binuclear copper ions so that the first copper ion demonstrated interactions with three histidine residues named His 61, His 85, and His95. The second copper ion coordinated by His 259, His 263, and His 296. Detailed and comprehensive studies confirmed that interactions with these critical residues can completely inactivate the monophenolase and diphenolase activity.

**Table 2:** Molecular docking results of compound **3f** with mushroom tyrosinase (PDB ID: 2Y9X)

Compound	Residues	Interaction type	Distance
	His259	Hydrogen bond	3.07
	Val283	Hydrogen bond	2.77
	Val283	Hydrophobic (Pi-Sigma)	3.80
	His296	Hydrogen bond	2.78
	Met280	Hydrogen bond	2.80
**3f**	Gly281	Hydrogen bond	3.74
	His263	Electrostatic (Pi-Cation)	3.88
	Arg268	Electrostatic (Pi-Cation)	3.76
	His263	Hydrophobic (Pi–Pi)	3.97
	Phe264	Hydrophobic (Pi–Pi)	5.24
	Ala286	Hydrophobic (Pi-Alkyl)	5.09

**Fig. 4:**
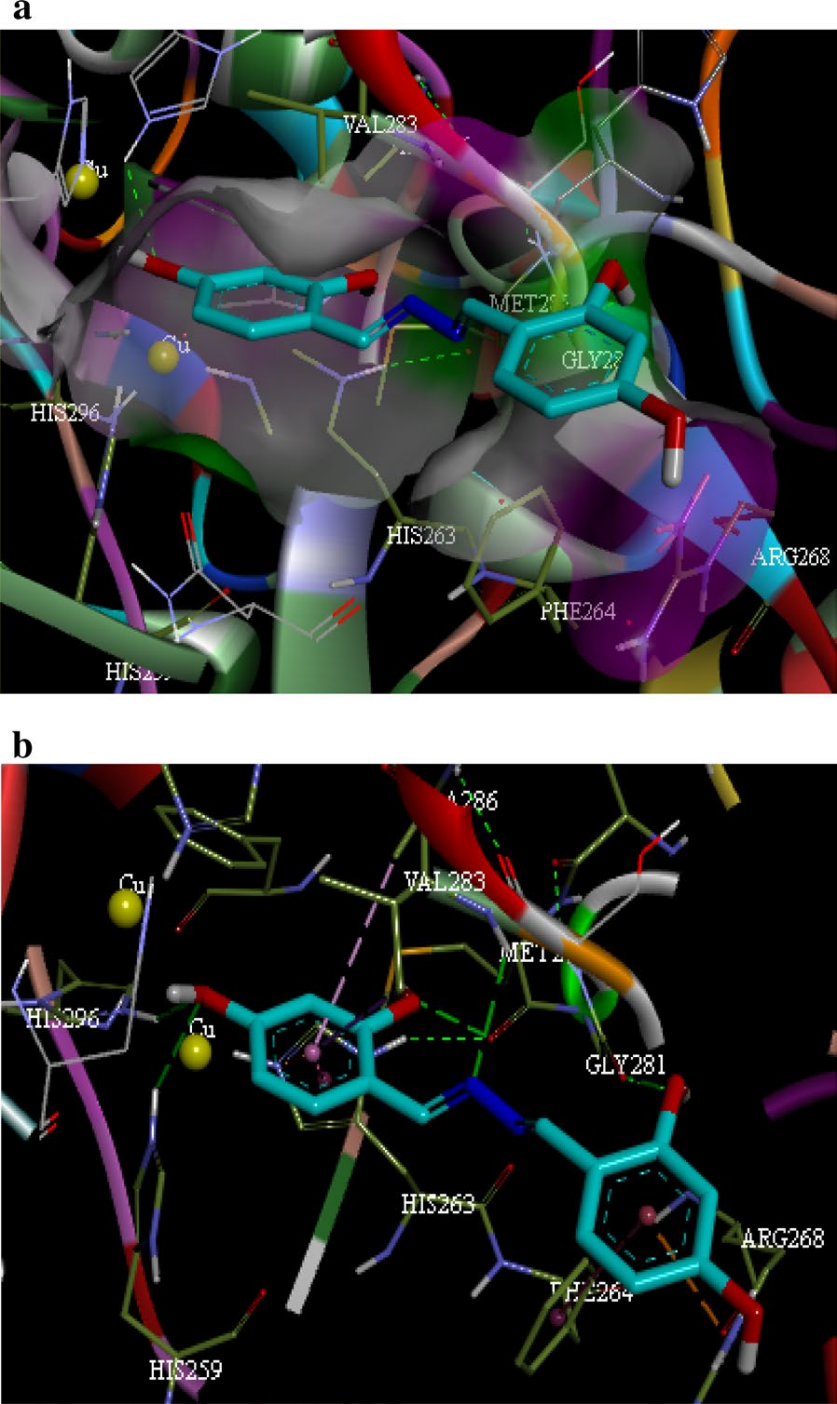
The binding orientation (**a**) and interactions (**b**) of compound **3f** into the tyrosinase enzyme. Ligand **3f** is displayed as cyan sticks, while the core residues are shown as green sticks. Hydrogen bonding, electrostatic, Pi–Pi, and alkyl-Pi interactions are displayed as green, orange, pink and light pink, respectively

As shown in Fig. 4, compound **3f** fitted well in the tyrosinase binding pocket by hydrogen bonding, electrostatic and hydrophobic interactions. The nitrogen atoms and the oxygen atoms of **3f** formed five hydrogen bonds interacting with the Val 283, Gly 281, His 296, Met 280, and His 259 residues at distances of 2.80 Å, 3.74 Å, 2.78 Å, 2.80 Å, and 3.07 Å, respectively. Moreover, compound **3f** was involved in electrostatic interactions with His263 and Arg268 as well as hydrophobic interactions with Phe264, Val283, His263, and Ala286. Docking results indicated that the substitution on the phenyl ring played an important role in forming drug-receptor interactions and binding orientation of the compound in the active site of tyrosinase by enhancing phenyl ring electron density.

### Cell viability on B16F10

MTT assay was performed to determine the toxicity of **3f** as the most potent tyrosinase inhibitor against invasive melanoma B16F10 cell line. According to the toxicity assay, **3f** showed 60.15 ± 9.82% (± SD) cell viability at 10 µM.

### Measuring cell viability of A375 in response to 3f

The A375 cell line is derived from a human skin melanoma carrying two mutant genes, B-RAF and CDKN2 which are associated with melanoma of sun-damaged skin [48]. To determine the cytotoxicity of **3f** in A375 melanoma cell lines, MTT studies were conducted. Results exhibited that the cell viability was significantly higher in A375 with 82.59 ± 5.85 (± SD) % viability compared with the B16F10 cell line at 10 µM.

### Melanin content assay

**Fig. 5:**
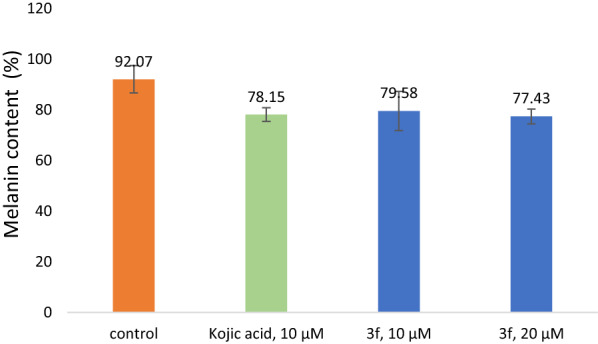
Effect of compound **3f** on melanin content in B16F10 melanoma cells

It is well documented that effective tyrosinase inhibitors may reduce the phenolase activities of the enzyme which in return downregulates the melanogenesis process [49, 50]. In this regard, the potency of **3f** to reduce the melanin content was evaluated on the B16F10 cell line. Kojic acid was used as the positive control. Data were expressed as mean ± SD of at least three independent experiments. According to Fig. 5, **3f** significantly reduced the melanin content in skin melanoma cells to 79.58% at 10 µM in comparison with the control (P-value < 0.05).

## Conclusion

Following our expertise in the rational design of tyrosinase inhibitors; herein, we designed, synthesized, and evaluated different azine substituted derivatives against tyrosinase. The most active compound **3f** bearing 2,4-dihydroxy on the benzyl ring (IC_50_ = 7.30 ± 1.15 μM) showed 3 times better potency compared to that of kojic acid as the positive control. Moreover, it is worth mentioning that **3f** showed an uncompetitive inhibition mode of action in the enzymatic assay. Molecular docking analysis demonstrated that the high potential of **3f** would be due to the formation of strong interactions with the critical residues of the tyrosinase active site. In addition, cell assessments of **3f** at 10 µM against B16F10 and A375 cell lines exhibited around 60% and 82% viability, respectively. Importantly, compound **3f** showed approximately similar potency to reduce the melanin content on B16F10 cell lines at 10 µM compared to kojic acid as a positive control. In general, it can be concluded that the synthesized compounds can serve as structural outlines and promising lead to design and expand potential tyrosinase inhibitors.

## Material and methods

### General

All needed chemicals were purchased from Merck and Acros chemical companies. All reagents and solvents were dried before use according to standard methods. Mushroom tyrosinase (EC1.14.18.1), kojic acid, dimethyl sulfoxide (DMSO), and l-3,4-dihydroxyphenylalanine (L-DOPA) were purchased from Sigma Chemical Co. (St. Louis, MO, USA). IR spectra were obtained on an FT-IR Perkin-Elmer Precisely system spectrophotometer (potassium bromide disks). Melting points were determined on a Kofler hot stage apparatus and are uncorrected. ^1^H NMR and ^13^C NMR spectra were recorded in DMSO using a Bruker Avance DPX instrument (^1^H NMR 500 MHz, ^13^C NMR 125 MHz). Chemical shifts (*δ*) were reported in parts per million (ppm) downfield from TMS as an internal standard. All of the coupling constants (*J*) are in hertz. The mass spectra were run on an Agilent 6410 apparatus. Merck silica gel 60 F254 plates were used for analytical thin-layer chromatography (TLC). Preparative thin-layer chromatography was done with prepared glass-backed plates (20 * 20 cm^2^) using silica gel (Merck-Kieselgel 60 HF254, Art. 7739).

### Synthesis

#### The typical procedure for the synthesis of bis aryl hydrazide derivatives

A mixture of aryl aldehyde (1 mmol), and hydrazine hydrate (0.5 mmol) were refluxed in ethanol for 24 h. The reaction proceeding was monitored by TLC (*n*-hexane/ethyl acetate, 20:1 v/v). After completion of the reaction, the mixture was cooled to room temperature, the precipitated solid was isolated by filtration, all products were recrystallized from hot ethanol. Physical and spectral data of bis aryl hydrazide derivatives are given below [51–53].

#### 1,2-di(Benzylidene)hydrazine (3a)

Yellow solid; Isolated yield: 65%; m.p: 88–93 °C; I.R (KBr, cm^−1^): 3067, 3050, 3025, 3000, 2949, 1624, 1574, 1491, 1446. ^1^H NMR (300 MHz, DMSO-*d*_6_) *δ*_H_ (ppm): 8.58 (s, 2H), 7.98–7.81 (m, 4H), 7.55–7.32 (m, 6H). ^13^C NMR (75 MHz, DMSO-*d*_6_) *δ*_c_ (ppm): 156.30, 134.20, 130.89, 128.76, 128.31. Anal. Calcd. For C_14_H_12_N_2_: C: 80.74; H: 5.81; N: 13.45; Found. C: 80.99; H: 5.83; N: 13.48.

#### 1,2-bis(2-Methoxybenzylidene)hydrazine (3b)

Commercially available with CAS number of 17745-81-2. Yellow solid; isolated yield: 64%; m.p: 142–146 °C; I.R (KBr) *Ʋ*_max_ (cm^−1^): 3075, 3005, 2965, 2946, 2842, 1616, 1576, 1485, 1468, 1438, 1321, 1250. ^1^H NMR (300 MHz, DMSO-*d*_6_) *δ*_H_ (ppm): 8.94 (s, 2H), 7.98 (d, *J* = 7.3 Hz, 2H), 7.51 (t, *J* = 7.5 Hz, 2H), 7.15 (d, *J* = 7.5 Hz, 2H), 7.05 (t, *J* = 7.5 Hz, 2H), 3.89 (s, 6H). ^13^C NMR (75 MHz, DMSO-*d*_6_) *δ*_c_ (ppm): 56.27, 112.49, 121.19, 122.08, 127.00, 133.50, 157.01, 159.22. MS (m/z, %): 77.3(41), 91.3(90), 119.3(65), 131.3(26), 150.3(35), 161.3(13), 237.3(92), 268.3(100). Anal. Calcd. For C_16_H_16_N_2_O_2_: C: 71.62; H: 6.01; N: 10.44; Found. C: 72.92; H: 6.00; N: 10.45.

#### 1,2-bis(4-Methoxybenzylidene)hydrazine (3c)

Commercially available with CAS number of 2299-73-2. Yellow solid; isolated yield: 58%; m.p: 164–166 °C; I.R (KBr) *Ʋ*_max_ (cm^−1^): 3092, 3059, 2982, 2971, 2931, 2878, 1631, 1600, 1474, 1439, 1400, 1377, 1357. ^1^H NMR (300 MHz, DMSO-*d*_6_) *δ*_H_ (ppm): 3.83 (s, 6H), 7.06 (s, 4H), 7.82 (s, 4H), 8.64 (s, 2H). ^13^C NMR (75 MHz, DMSO-*d*_6_) *δ*_c_ (ppm): 55.86, 114.87, 127.03, 130.44, 160.96, 162.15. MS (m/z, %): 77.3(34), 91.3(18), 107.3(42), 121.3(24), 134.3(46), 148.3(26), 161.3(70), 240.4(16), 268.3(M^+^, 83). Anal. Calcd. For C_16_H_16_N_2_O_2_: C: 71.62; H: 6.01; N: 10.44; Found. C: 72.92; H: 6.00; N: 10.45.

#### 4,4′-(Hydrazine-1,2-diylidenebis(methaneylylidene))bis(2-ethoxyphenol) (3d)

White solid; isolated yield: 69%; m.p: 210–218 °C; I.R (KBr) *Ʋ*_max_ (cm^−1^): 3435, 3292, 3052, 2987, 2968, 2927, 2838, 1620, 1500, 1461, 1420, 1377, 1251. ^1^H NMR (300 MHz, DMSO-*d*_6_) *δ*_H_ (ppm): 1.37 (t, *J* = 5.4 H), 4.07 (q, *J* = 5.4 H), 6.90 (d, *J* = 7.5 Hz, 2H), 7.26 (d, *J* = 7.5 Hz, 2H), 7.44 (s, 2H), 8.57 (s, 2H), 9.63 (s, 2H). ^13^C NMR (7.5 MHz, DMSO-*d*_6_) *δ*_c_ (ppm): 15.16, 64.30, 111.92, 116.06, 123.76, 126.00, 147.57, 150.55, 161.06. MS (m/z, %): 163.3(19), 191.4(41), 272.4(39), 299.4(23), 328.3(M^+^, 100). Anal. Calcd. For C_18_H_20_N_2_O_4_: C: 65.84; H: 6.14; N: 8.53; Found. C: 66.62; H: 6.15; N: 8.52.

#### 1,2-bis(3,4,5-Trimethoxybenzylidene)hydrazine (3e) [51]

Yellow solid; isolated yield: 75%; m.p: 201–205 °C; I.R (KBr) *Ʋ*_max_ (cm^−1^): 3083, 3063, 3012, 2989, 2955, 2936, 2840, 1623, 1505, 1457, 1434, 1457, 1375, 1150. ^1^H NMR (300 MHz, DMSO-*d*_6_) *δ*_H_ (ppm): 3.74 (s, 6H), 3.85 (s, 12H), 7.22 (s, 4H), 8.66 (s, 2H). ^13^C NMR (75 MHz, DMSO-*d*_6_) *δ*_c_ (ppm): 56.39, 60.63, 106.03, 129.73, 140.65, 153.63, 161.72. MS (m/z, %): 221.4(25), 345.4(23), 388.4(M^+^, 100). Anal. Calcd. For C_20_H_24_N_2_O_6_: C: 61.85; H: 6.23; N: 7.21; Found. C: 69.76; H: 6.24; N: 7.20.

#### 4,4′-(Hydrazine-1,2-diylidenebis(methaneylylidene))bis(benzene-1,3-diol) (3f)

Yellow solid; isolated yield: 92%; m.p: 313 °C; I.R (KBr) *Ʋ*_max_ (cm^−1^): 3518, 3468, 3214, 3097, 3096, 2998, 2936, 2891, 1616, 1589, 1534, 1453, 1251, 1121. ^1^H NMR (300 MHz, DMSO-*d*_6_) *δ*_H_ (ppm): 6.35 (d, *J* = 2.4 Hz, 2H), 6.41 (dd, *J* = 8.4, 2.4 Hz, 2H), 7.41 (d, *J* = 8.4 Hz, 2H), 8.77 (s, 2H), 10.27 (s, 2H), 11.39 (s, 2H). ^13^C NMR (75 MHz, DMSO-*d*_6_) *δ*_c_ (ppm): 102.95, 108.69, 110.73, 133.46, 161.16, 162.27, 162.54. MS (m/z, %): 137.3(79), 255.4(36), 272.2(M^+^, 100). Anal. Calcd. For C_14_H_12_N_2_O_4_: C: 61.76; H: 4.44; N: 10.29; Found. C: 60.88; H: 4.45; N: 10.28.

#### 1,2-bis(3,4-Dimethoxybenzylidene)hydrazine (3g) [52]

Commercially available with CAS number of 17745-86-7. Yellow solid; isolated yield: 94%; m.p: 197–199 °C; I.R (KBr) *Ʋ*_max_ (cm^−1^): 3079, 3003, 2962, 2930, 2840, 1624, 1580, 1542, 1465, 1443, 1345, 1259. ^1^H NMR (300 MHz, DMSO-*d*_6_) *δ*_H_ (ppm): 3.83 (s, 12H), 7.05 (s, 2H), 7.36 (s, 2H), 7.49 (s, 2H), 8.63 (s, 2H). ^13^C NMR (75 MHz, DMSO-*d*_6_) *δ*_c_ (ppm): 55.86, 56.09, 109.52, 111.95, 123.97, 127.16, 134.76, 149.46, 161.25. MS (m/z, %): 79.2(23), 164.3(13), 191.3(52), 286.4(27), 313.4(9), 328.3(M^+^, 100). Anal. Calcd. For C_18_H_20_N_2_O_4_: C: 65.84; H: 6.14; N: 8.53; Found. C: 64.96; H: 6.15; N: 8.52.

#### 5,5′-(Hydrazine-1,2-diylidenebis(methaneylylidene))bis(2-methoxyphenol)(3h) [53]

Yellow solid; isolated yield: 73%; m.p: 270–275 °C; I.R (KBr) *Ʋ*_max_ (cm^−1^): 3295, 3074, 3009, 2941, 2842, 1619, 1579, 1508, 1461, 1439, 1360, 1273, 1027. ^1^H NMR (300 MHz, DMSO-*d*_6_) *δ*_H_ (ppm): 3.83 (s, 6H), 7.01 (d, *J* = 8.1 Hz, 2H), 7.23 (d, *J* = 8.1 Hz, 2H), 7.38 (s, 2H), 8.53 (s, 2H), 9.36 (s, 2H). ^13^C NMR (75 MHz, DMSO-*d*_6_) *δ*_c_ (ppm): 56.05, 112.25, 113.78, 122.29, 127.33, 147.23, 151.08, 161.13. MS (m/z, %): 150.3(12), 177.3(64), 273.3(32), 300.3(M^+^, 100). Anal. Calcd. For C_16_H_16_N_2_O_4_: C: 63.99; H: 5.37; N: 9.33; Found. C: 63.79; H: 5.39; N: 9.38.

#### (Hydrazine-1,2-diylidenebis(methaneylylidene))bis(2-methoxy-4,1-phenylene) diacetate (3i)

Yellow solid; isolated yield: 53%; m.p: 165–170 °C; I.R (KBr) *Ʋ*_max_ (cm^−1^): 3075, 3010, 2944, 2887, 2849, 1765, 1632, 1599, 1508, 1474, 1457, 1375, 1272, 1155, 1030, 1010. ^1^H NMR (300 MHz, DMSO-*d*_6_) *δ*_H_ (ppm): 2.29 (s, 6H), 3.86 (s, 6H), 7.25 (d, *J* = 7.2 Hz, 2H), 7.47 (d, *J* = 7.2 Hz, 2H), 7.65 (s, 2H), 8.73 (s, 2H). ^13^C NMR (75 MHz, DMSO-*d*_6_) *δ*_c_ (ppm): 20.86, 56.33, 111.54, 122.45, 123.89, 133.11, 142.32, 151.70, 161.36, 168.81. MS (m/z, %): 43.3(57), 177.3(34), 258.4(12), 273.4(23), 300.4(100), 341.5(51), 384.3(M^+^, 79). Anal. Calcd. For C_20_H_20_N_2_O_6_: C: 62.49; H: 5.24; N: 7.29; Found. C: 62.47; H: 5.25; N: 7.32.

#### 4,4′-(Hydrazine-1,2-diylidenebis(methaneylylidene))diphenol (3j)

Yellow solid; isolated yield: 78%; m.p: 204–208 °C; I.R (KBr) *Ʋ*_max_ (cm^−1^): 3367, 3069, 3012, 2988, 2955, 2839, 1620, 1577, 1490, 1470, 1280, 1089. ^1^H NMR (300 MHz, DMSO-*d*_6_) *δ*_H_ (ppm): 6.87 (d, *J* = 8.4 Hz, 4H), 7.70 (d, *J* = 8.4 Hz, 4H), 8.56 (s, 2H), 10.11 (s, 2H). ^13^C NMR (75 MHz, DMSO-*d*_6_) *δ*_c_ (ppm): 116.22, 125.57, 130.56, 160.75, 160.84. MS (m/z, %): 65.3(68), 93.3(22), 120.3(40), 147.3(84), 212.3(27), 223.3(9), 240.3(M^+^, 100). Anal. Calcd. For C_14_H_12_N_2_O_2_: C: 69.99; H: 5.03; N: 11.66; Found. C: 69.76; H: 5.09; N: 11.69.

#### 4,4′-(Hydrazine-1,2-diylidenebis(methaneylylidene))bis(2-methoxyphenol) (3k)

Commercially available with CAS number of 1696-60-2, Yellow solid; isolated yield: 62%; m.p: 169–174 °C; I.R (KBr) *Ʋ*_max_ (cm^−1^): 3481, 3084, 3003, 2958, 2937, 2921, 2856, 1625, 1602, 1509, 1453, 1427, 1239, 1032. ^1^H NMR (300 MHz, DMSO-*d*_6_) *δ*_H_ (ppm): 3.84 (s, 6H), 6.89 (d, *J* = 8.4 Hz, 2H), 7.27 (dd, *J* = 8.4, 1.8 Hz, 2H), 7.47 (d, *J* = 1.8 Hz, 2H), 8.58 (s, 2H), 9.72 (s, 2H). ^13^C NMR (75 MHz, DMSO-*d*_6_) *δ*_c_ (ppm): 55.97, 110.48, 115.95, 123.96, 125.99, 148.44, 150.34, 161.08. MS (m/z, %): 150.3(12), 177.3(54), 258.4(25), 272.5(18), 299.6(54), 300.3(M^+^, 100). Anal. Calcd. For C_16_H_16_N_2_O_4_: C: 63.99; H: 5.37; N: 9.33; Found. C: 63.79; H: 5.39; N: 9.38.

### Tyrosinase assay

Mushroom tyrosinase (EC 1.14.18.1) (Sigma Chemical Co.) was assayed as explained previously with slight modifications applying L-DOPA as substrate [54]. In spectrophotometric experiments, enzyme activity was monitored by observing dopachrome formation at 475 nm. The stock solutions of test compounds **3a**–**k** and kojic acid were first dissolved in DMSO at 40 mM and then diluted with phosphate buffer (pH = 6.8) to the required concentrations. First, 10 µL of mushroom tyrosinase (0.5 mg mL^−1^) was mixed with 160 µL of phosphate buffer (50 mM, pH = 6.8) and then 10 µL of the test sample in 96-well microplates was added. After the mixture was pre-incubated at 28 °C for 20 min, 20 µL of L-DOPA solution (0.5 mM) was added to the phosphate buffer and dopachrome formation was monitored at 475 nm for 10 min. DMSO without test compounds was used as the control, and kojic acid was used as the positive control. Each assay was conducted as three separate replicates. The final concentration of DMSO in the test solution was less than 2.0%. The percent inhibition ratio was calculated according to the following equation:

$$ {\text{Inhibition}}\left( \% \right) = 100 \times {{\left( {{\text{Abs}}_{{{\text{control}}}} - {\text{Abs}}_{{{\text{compound}}}} } \right)} \mathord{\left/ {\vphantom {{\left( {{\text{Abs}}_{{{\text{control}}}} - {\text{Abs}}_{{{\text{compound}}}} } \right)} {{\text{Abs}}_{{{\text{control}}}} }}} \right. \kern-\nulldelimiterspace} {{\text{Abs}}_{{{\text{control}}}} }}. $$

The inhibitory activity of the tested compounds was expressed as the concentration that inhibited 50% of the enzyme activity (IC_50_).

### Determination of the inhibition type

A series of experiments were performed to determine the inhibition kinetics of **3f**. The inhibitor concentrations were: 0, 8, 16 μM. Substrate (L-DOPA) concentrations were (0.1, 0.25, 0.5, 0.75 and 1.5 mM) in kinetic studies. Pre-incubation and measurement time was the same as discussed in the mushroom tyrosinase inhibition assay protocol. Maximum initial velocity was determined from the initial linear portion of absorbance up to 10 min after addition of L-DOPA with 1 min interval. The Michaelis constant (K_m_) and the maximal velocity (V_max_) of the tyrosinase activity were determined by the Lineweaver–Burk plot at various concentrations of L-DOPA as a substrate. The inhibition type of the enzyme was assayed by Lineweaver–Burk plots of the inverse of velocities (1/V) versus the inverse of substrate concentrations 1/[S] mM^−1^.

### Molecular docking study

Docking was done by AutoDock 4.2 (http://autodock.scripps.edu) and AutoDock Tools 1.5.4 (ADT) (http://mgltools.scripps.edu/). X-ray crystal structure of agaricus bisporus tyrosinase containing tropolone in the active site (PDB ID: 2Y9X), was regained from protein data bank (http://www.rcsb.org). Before the docking method, the water molecules and the inherent ligand were eliminated from the protein. Hydrogens were attached and non-polar hydrogens were merged. Also, Gasteiger charges were calculated for protein 2Y9X. 3D structures of ligands were drawn and minimized under Molecular Mechanics MM^+^ and then Semi-empirical AM1 methods using HyperChem software (http://www.hyper.com/). The pdbqt formats of the ligands were prepared by adding Gasteiger charges and setting the degree of torsions. The active site which contains Cu^2+^ metal ions were chosen for docking and the grids’ center was placed on the tropolone’s binding site. The box dimensions were set to 40 × 40 × 40 with 0.375 Å grid spacing. To determine the docking parameter file, a rigid macromolecule was elected. The Lamarckian genetic search algorithm was used and the number of GA runs was determined at 100. The other parameters were left at program default values. The validity of the docking procedure was tested using the co-crystallized inhibitor as ligand and the above-mentioned protocol. Finally, conformations having the lowest assumed free energies of binding were studied to be analyzed [55].

### MTT assay for cell viability

The cytotoxic activity of compound was evaluated in B16F10 cells using 3-(4,5-dimethylthiazol-2-yl)-2,5-diphenyltetrazolium bromide (MTT) assay. Cells were seeded in a 96-well plate (5 × 10^3^ cells/well) and incubated at 37 °C with samples at different concentrations for 48 h. Following the treatment, cells were incubated with MTT (0.5 mg mL^−1^) at 37 °C for 3 h. The MTT-containing medium was then removed, and 100 μL of DMSO was added to each well, mixed thoroughly with a 10 min shake to dissolve formazan crystals. The absorbance of each well was measured at 540 nm.

### Determination of melanin content

B16F10 cells were seeded in six-well plates (1.0 × 10^5^ cells/well). After 24 h, the medium was substituted by a fresh one and treated with **3f**, and incubated for 48 h. Then, cells were treated with 100 nM $\alpha$-MSH. Kojic acid was used as positive control and for comparing the inhibitory strength of the compound. After incubation cells were washed twice with PBS and harvested using 0.25 M trypsin, then dissolved in 300 μL of 1 N NaOH/10% DMSO buffer and boiled for 2 h at 80 °C to solubilize the melanin. The absorbance of the supernatant was measured at 470 nm in a microplate reader. The obtained results were normalized using total protein content.

## Data Availability

The datasets used and analyzed during the current study are available from the corresponding author on reasonable request.
